# New Hampshire Veterinary Medical Association

**Published:** 1895-06

**Authors:** L. Pope

**Affiliations:** Secretary


### NEW HAMPSHIRE VETERINARY MEDICAL ASSOCIATION.

ON May 7th, veterinarians of New Hampshire met in the Eagle Hotel, Concord, and organized a State Veterinary Association. The Constitution and by-laws were signed, having been framed and accepted at a previous meeting in April. Officers for the ensuing year were elected as follows: *President*, W. Russell, Nashua; *Vice-President*, J. Hart, Concord; *Secretary*, L. Pope, Jr., Portsmouth; *Treasurer*, A. F. Abbott, Manchester; *Executive Committee*, Drs. L. E. Tuttle, Franklin Falls; A. L. Dodge, Manchester; R. J. Macguire, Concord. L. POPE, JR.,

Secretary.

